# Loss of PDK4 expression promotes proliferation, tumorigenicity, motility and invasion of hepatocellular carcinoma cells

**DOI:** 10.7150/jca.43459

**Published:** 2020-05-18

**Authors:** Yu-Juan Qin, Tao-Yan Lin, Xiao-Lin Lin, Yu Liu, Wen-Tao Zhao, Xiao-Yan Li, Mei Lian, Heng-Wei Chen, Yong-Long Li, Xiao-Ling Zhang, Dong Xiao, Jun-Shuang Jia, Yan Sun

**Affiliations:** 1 Guangdong Provincial Key Laboratory of Cancer Immunotherapy Research and Guangzhou Key Laboratory of Tumor Immunology Research, Cancer Research Institute, School of Basic Medical Sciences, Southern Medical University, Guangzhou 510515, China; 2 Zhongshan School of Medicine, Sun Yat-sen University, Guangzhou 510080, China; 3 Institute of Comparative Medicine & Laboratory Animal Center, Southern Medical University, Guangzhou 510515, China; 4 Department of Radiology, The 5th Affiliated Hospital of Sun Yat-Sen University, Zhuhai 519000, China; 5 Department of Pharmacy, Nanfang Hospital, Southern Medical University, Guangzhou 510515, China; 6 Department of Medical Oncology, The Third Affiliated Hospital of Kunming Medical University (Tumor Hospital of Yunnan Province), Kunming 650118, China; 7 Department of Physiology, Faculty of Basic Medical Sciences, Guilin Medical University, Guilin 541004, China

**Keywords:** Hepatocellular carcinoma, Pyruvate dehydrogenase kinase 4, Cell proliferation, Migration, Invasion

## Abstract

Although the roles and underlying mechanisms of other PDK family members (i.e., PDK1, PDK2 and PDK3) in tumor progression have been extensively investigated and are well understood, the functions and underlying molecular mechanisms of pyruvate dehydrogenase kinase 4 (PDK4) in the tumorigenesis and progression of various cancers [including hepatocellular carcinoma (HCC)] remain largely unknown. In this study, we examined the expression profile of PDK4 in HCC clinical tissue specimens and the roles of PDK4 in the proliferation, tumorigenicity, motility and invasion of HCC cells. The immunohistochemistry (IHC) and quantitative real-time PCR (qRT-PCR) results revealed that PDK4 was significantly downregulated in the cohort of HCC clinical specimens. Additionally, PDK4 protein was found in both the nucleus and cytoplasm of HCC cells based on an immunofluorescence (ICC) assay, and PDK4 protein was also found in the nucleus and cytoplasm of cancer cells contained in HCC clinical specimens based on IHC. The CCK-8 assay and cell colony formation assay demonstrated that stable depletion of endogenous PDK4 by lentivirus-mediated RNA interference (RNAi) markedly promoted the proliferation of HCC cell lines (i.e., BEL-7402 and BEL-7404 cells) in vitro, while PDK4 silencing significantly enhanced the tumorigenic ability of BEL-7404 cells in vivo. In addition to enhance proliferation and tumorigenesis induced by PDK4 silencing, additional studies demonstrated that knockdown of PDK4 led to increase migration and invasion of BEL-7402 and BEL-7404 cells in vitro. Taken together, these findings suggest that the loss of PDK4 expression contributes to HCC malignant progression.

## Introduction

Hepatocellular carcinoma (HCC) is the fifth most common and the third most deadly cancer worldwide with an extremely poor prognosis 1-7. HCC is particularly prevalent in China, Southeast and Eastern Asia and sub-Saharan Africa, and in China, the development of HCC is highly associated with hepatitis B virus (HBV) infection 1-7. Because of the lack of effective therapy and early detection, HCC patients have a poor 5-year survival rate; research aimed at addressing the molecular mechanisms of HCC pathogenesis should help develop new tools for early-stage diagnosis and effective therapy 1-7.

The pyruvate dehydrogenase complex (PDC), a key regulator of tricarboxylic acid (TCA) cycle flux, catalyzes oxidative conversion of pyruvate into acetyl CoA and NADH in mitochondria, which is required for the TCA cycle and mitochondrial respiration, while phosphorylation of PDC is catalyzed in humans by any of four isozymes of the pyruvate dehydrogenase kinase (PDK1, PDK2, PDK3 and PDK4), which exhibit 70% homology 8-13. Accumulating evidence has shown that PDK1-3 are closely associated with the metabolism of tumor cells because they can phosphorylate PDC, leading to the inactivation of PDC 8-13. PDK1-3 are universally overexpressed in various cancer cells, including multiple myeloma, HCC and malignant glioma, which has led to the idea of utilizing PDKs as therapeutic targets for the treatment of cancers, while the successful development of highly potent inhibitors of PDK1-3 can provide a powerful approach for killing tumor cells or, at least, greatly reducing tumor cell growth 8-13.

PDK4 is predominantly expressed in the heart, skeletal muscle, kidneys and pancreatic islets 8-13. There are several lines of evidence that indicate that PDK4 is involved in cancer progression 14-20. These findings from microarray data 17, 19 and qRT-PCR 14 have revealed that PDK4 mRNA expression is dramatically decreased in multiple human cancers, including breast, ovarian, colon, and lung cancers. PDK4 inhibition via RNA interference (RNAi)-mediated knockdown drove epithelial-mesenchymal transition (EMT) and promoted erlotinib resistance in EGFR mutant lung cancer cells 19, and siPDK4 in ovarian cancer cells promoted EMT and invasion but the effect was attenuated by PDK4 overexpression 20. Conversely, PDK4 silencing by RNAi decreased the migration, invasion and resistance to apoptosis of colon cancer cells 15. Additionally, miR-182 is dysregulated and inversely correlated with PDK4 in human lung adenocarcinomas, and miR-182 suppressed PDK4 expression and promoted lung tumorigenesis 16. In summary, unlike the roles of PDK1-3, the roles of PDK4 in tumor progression and the underlying molecular mechanisms remain largely unclear. In the present study, we investigated whether PDK4 is involved in the proliferation, tumorigenicity, motility and invasion of HCC cells.

## Materials and methods

### Clinical specimens

Thirty-nine paired specimens of HCC and adjacent noncancerous liver tissues and 61 HCC specimens were collected from Sun Yat-sen Memorial Hospital, Sun Yat-sen University, Guangzhou with informed consent following the institutional-review-board-approved protocols. The inclusion criteria of HCC cases were as follows: (1) a clear pathological diagnosis of HCC, (2) no anticancer treatment before surgery, (3) suitable formalin-fixed, paraffin-embedded tissues, and (3) complete clinicopathologic and follow-up data. Tumor stage was defined according to the 2009 American Joint Committee on Cancer/International Union Against Cancer tumor-node-metastasis classification system. The histological grade of tumor differentiation was determined by the Edmondson grading system 23. Ethical approval was given by the Medical Ethics Committee of Southern Medical University, with the following reference number: 2017-002-01.

### Histological analysis and immunohistochemistry (IHC)

For histological analysis, human tumor xenografts in nude mice, human HCC clinical specimens and adjacent non-cancerous liver tissues were collected, fixed in 4% phosphate-buffered paraformaldehyde (PFA) at 4℃ overnight, embedded in paraffin, and then cut into 5 mm thick sections. Subsequently, the tissue sections were mounted on slides, dewaxed and then deparaffinized. Hematoxylin and eosin staining (H&E staining) was subsequently carried out according to standard procedures.

The immunohistochemical staining procedure followed the standard streptavidin-peroxidase (SP) protocol. After deparaffinization and rehydration, the paraffin-embedded sections were subjected to high-pressure treatment in citrate buffer (pH 6.0) and boiling for 2 mins for antigenic retrieval. Endogenous peroxidase and non-specific staining were blocked by with H_2_O_2_ and 1% BSA for 15 mins at room temperature, respectively. The sections were then incubated overnight at 4℃ with the primary antibodies against PDK4 (Proteintech, dilution 1:250), BrdU (GE Healthcare, dilution 1:50) or Ki67 (Abcam, dilution 1:250). PBS was used as negative controls. The complex was visualized with DAB and counterstained with hematoxylin.

Low and high expression of PDK4 were defined by previously described standards 21. The staining intensity of tumor cells was grouped into four grades: 0, no staining; 1, weak staining; 2, modest staining; and 3, strong staining. The positive staining ratio of tumor cells was classified into four grades: 0, no positive tumor cells; 1, < 10% positive tumor cells; 2, 10-50% positive tumor cells; and 3, > 50% positive tumor cells. The positive staining ratio of tumor cells = PDK4-positive tumor cells/total tumor cellsX100%. The general IHC results were calculated by multiplying the positive staining grade by the intensity grade (0, 1, 2, 3, 4, 6, and 9). Finally, general IHC results ≤4 and ≥6 were defined as low and high expression, respectively. Two pathologists examined and scored IHC results blindly without knowing the clinical characteristics and prognosis.

### RNA isolation and quantitative real-time PCR (qRT-PCR)

RNA isolation, reverse transcription and qRT-PCR were performed as previously described 21. The sequences were as follows: PDK4 forward primer: 5'-AACACCAGGAAAATCAGCC-3'; PDK4 reverse primer: 5'-AAAACCAGCCAAAGGAGC-3'; GAPDH forward primer: 5'-ACCCAGAAGACTGTGGATGG-3'; and GAPDH reverse primer: 5'-TCTAGACGGCAGGTCAGGTC-3'. GAPDH was used as an endogenous control. All samples were normalized to internal controls, and fold changes were calculated through relative quantification (2^-△△Ct^).

### Immunofluorescence assay (ICC)

For immunofluorescence assay, BEL-7402 and BEL-7404 cells (i.e., 7402 and 7404 cells) grown on the surface of cover slides, were fixed with 4% paraformaldehyde, rehydrated, and incubated with rabbit anti-PDK4 at room temperature for 40 mins. Subsequently, cells were incubated with goat anti-rabbit IgG(H+L) Dylight 549 (1:1000, Bioworld Technology, Inc.) for 40 mins at room temperature. The nuclei were stained with 4',6-diamidino-2-phenylindole (DAPI). Slides were examined with the ECLIP SE 80i fluorescent microscope (Nikon, Japan).

### Cell lines and cell cultures

Human HCC cell lines, including 7402 and 7404 cells, were purchased from the Type Culture Collection of the Chinese Academy of Sciences, Shanghai, China. HEK293T cells were purchased from the American Type Culture Collection (ATCC). All cells were approved by the Institutional Review Board of Southern Medical University. All cells were cultured in Dulbecco's modified Eagle's medium (DMEM) (Corning) supplemented with 10% fetal bovine serum (FBS) (Biological Industries) in a humidified incubator with 5% CO_2_ at 37°C.

### Plasmids, lentivirus production and lentiviral transduction for stable cell lines

Four lentiviral short-hairpin RNA (shRNA) constructs for human PDK4 (Cat. No: i017043) and a lentiviral scrambled control shRNA (shSCR) construct (Cat. No: LV015-G) were obtained from Applied Biological Materials (ABM) Inc. (Canada). The lentiviral vectors were cotransfected with the lentiviral packaging plasmids psPAX2 and pMD2G (Addgene) into 293T cells for lentivirus production as previously described^22^, and subsequently, the above lentiviruses were used to infect 7402 and 7404 cells.

### Western blotting analysis

Protein lysates were separated by sodium dodecyl sulfate-polyacrylamide gel electrophoresis (SDS-PAGE) and transferred to a polyvinylidene difluoride (PVDF) membrane. The blots were probed with primary antibodies against βactin (Cell Signaling, rabbit, 1:1000 dilution) or PDK4 (Proteintech, rabbit, 1:1000 dilution), followed by HRP (horseradish peroxidase)-labeled secondary antibodies. The hybridization signal was detected by enhanced chemiluminescence (ECL) (Cat. No: KGP1122, KeyGEN BioTECH). βactin was used as a loading control.

### CCK-8 assay and colony formation assay

The CCK-8 and colony formation assays were performed as previously described 25. For the CCK-8 assay (Cat. No: CK04, Dojindo, Japan), 7402 and 7404 cells stably expressing shSCR or shPDK4 were plated in 96-well plates (1×10^3^ cells/well) in a final volume of 200μl and then cultured for 7 days. For the colony formation assay, cells were counted and plated at 200 cells/well in six-well plates for 14 days.

### Transwell migration assay and Boyden invasion assay

Transwell migration and boyden invasion assays were performed as previously described 24. For the transwell migration assay, shSCR- or shPDK4-expressing 7402 and 7404 cells (1×10^5^) were seeded into the upper chamber (BD Biosciences, MA) with serum-free DMEM. A boyden invasion assay was conducted with matrigel (BD Biosciences) in the upper chamber. DMEM with 10% FBS was added into the lower compartment as a chemoattractant. Cells were allowed to migrate for 14 h and 20 h in the transwell migration assay and boyden invasion assay, respectively.

### Tumor xenografts in animals

Male BALB/c nude mice (3-4 weeks) were purchased from the Medical Laboratory Animal Center of Guangdong Province and were fed autoclaved water and laboratory rodent chow. The 7404 cells that stably expressed shSCR (1×10^6^ cells) or shPDK4 (1×10^6^ cells) were subcutaneously injected into the left or right dorsal thigh of the mice (n=6). The animals were monitored daily, and tumor volumes were measured every 2-3 days using a caliper slide rule. Tumor volume was determined using the following formula: volume =1/2 (width^2^ × length) 0.5 × width^2^ × length. All animals were sacrificed on the fourteenth day after transplantation. This animal experiment was carried out in strict accordance with the recommendations in the Guide for the Care and Use of Laboratory Animals of the Southern Medical University. The animal protocol was approved by the Committee on Ethics of Animal Experiments of the Southern Medical University (2016001).

### Statistical analysis

Data are presented as the mean ± SD from three independent experiments. Statistical analysis was performed using SPSS 16.0 software (SPSS, North Chicago, IL). Statistical significance was assessed by Student's t-test (*P< 0.05, **P< 0.01 and ^#^*P*< 0.001).

## Results

### Reduced PDK4 expression is frequently detected in HCC tissue specimens

**Fig 1:**
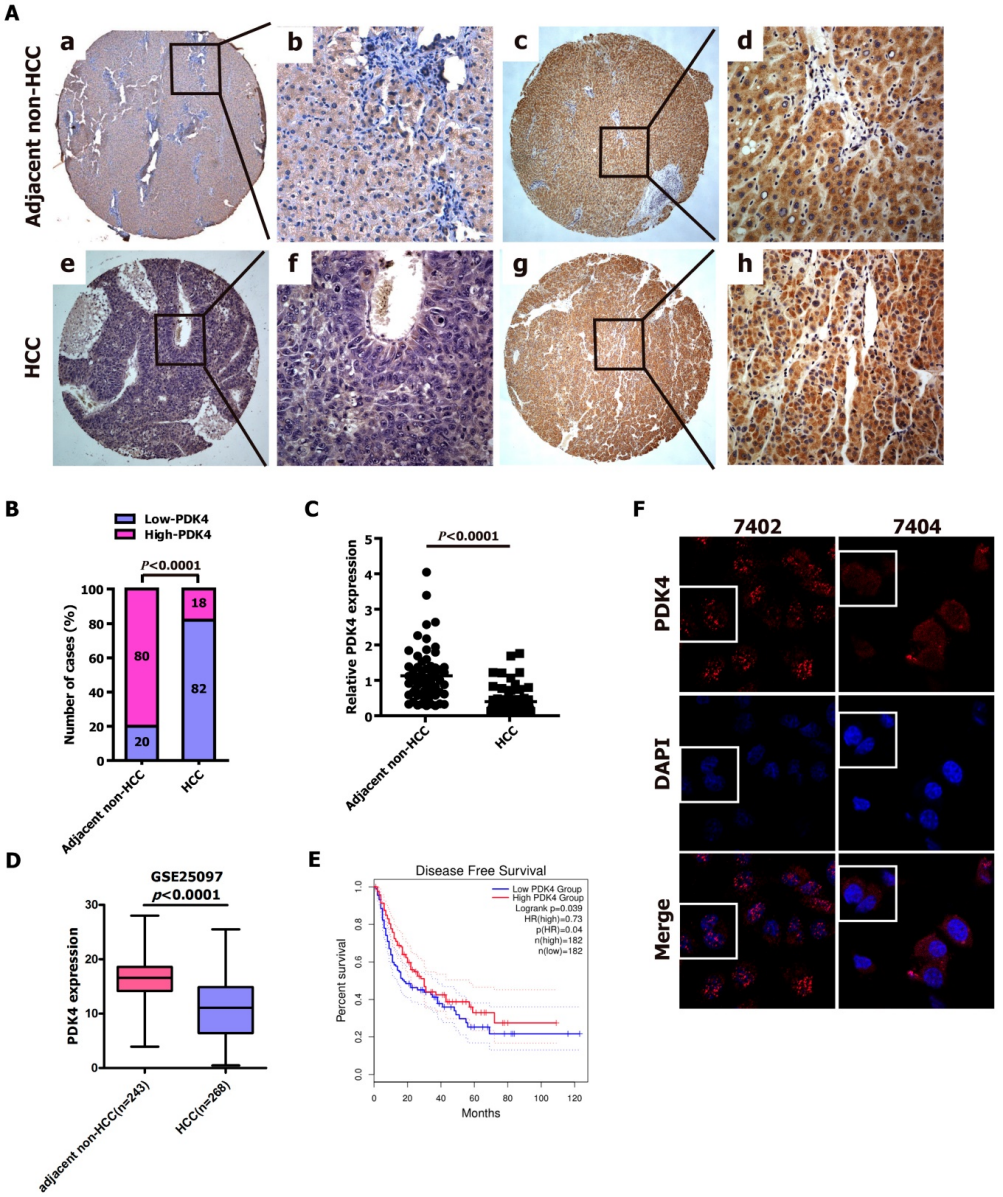
** PDK4 expression level was significantly lower in HCC tissue specimens than that in adjacent non-cancerous liver tissues. (A)** Representative photographs of PDK4 protein expression levels in adjacent non-cancerous liver specimens and HCC tissue specimens examined by IHC. a and b: Low expression of PDK4 in adjacent non-cancerous liver tissues; c and d: High expression of PDK4 in adjacent non-cancerous liver tissues; e and f: Low expression of PDK4 in HCC specimens; g and h: High expression of PDK4 in HCC specimens. The brown staining indicates PDK4 immunoreactivity. **(B)** IHC assay revealed that PDK4 expression level was significantly lower in the HCC tissue specimens than that in the adjacent non-cancerous liver tissues (*P*<0.0001, χ^2^ test). **(C)** The transcript levels of PDK4 were detected in HCC tissue specimens (n=61) and their corresponding adjacent non-cancerous liver tissues (n=61) by qRT-PCR (*P*<0.0001, Student's t-test). **(D)** The expression levels of PDK4 in HCC tissue specimens (n=268) and adjacent non-cancerous liver tissues (n=243) from GEO datasets. **(E)** Kaplan-Meier analysis of overall survival time in HCC tissue specimens based on PDK4 expression from GEPIA database. **(F)** Immunofluorescence assay indicated that the distribution of PDK4 protein in cytoplasm and nucleus of 7402 and 7404 cells. The nuclei was stained with DAPI.

**Fig 2:**
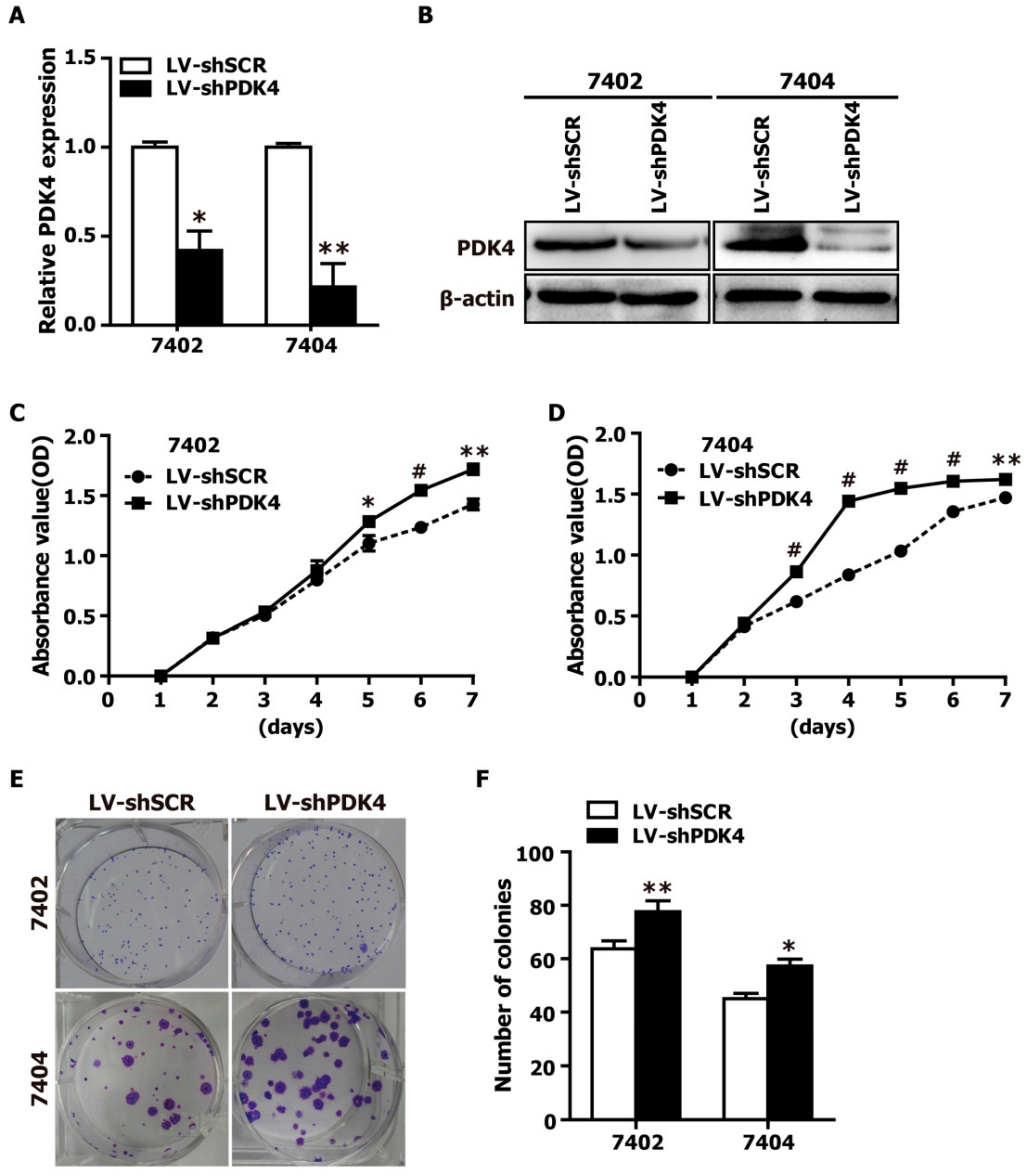
** RNAi-mediated silencing of PDK4 promoted the proliferation of HCC cells* in vitro*.** (**A**) The relative mRNA levels of PDK4 in shPDK4-expressing 7402 and 7404 cells based on qRT-PCR assay. SCR: scrambled control shRNA. (**B**) The protein levels of PDK4 in shPDK4-expressing 7402 and 7404 cells based on western blot analysis. (**C-D**) The CCK-8 assay was used to evaluate the proliferation of the shSCR- and shPDK4-expressing 7402 (C) and 7404 cells (D). (**E-F**) Colony formation assay was performed to test the proliferation ability of the shSCR- and shPDK4-expressing 7402 and 7404 cells. Statistical significance was assessed by Student's t-test (*P< 0.05, **P< 0.01 and ^#^*P*< 0.001).

To explore whether PDK4 is involved in the progression of HCC, we first evaluated the expression of the PDK4 protein in 39 paired paraffin-embedded, archived specimens of HCC and adjacent noncancerous liver tissues using IHC staining. The results from IHC staining revealed that low expression of PDK4 was detected in 8 out of 39 adjacent non-cancerous liver tissues (20.5%)(Fig. 1A-a,b,c,d and Supplementary Table 1) and 32 out of 39 HCC specimens (82.1%)(Fig. 1A-e,f,g,h and Supplementary Table 1). The statistical results were also shown in Fig. 1B and Supplementary Table 1. Additionally, we quantitatively evaluated PDK4 expression in 61 HCC biopsies using qRT-PCR, and we found that the expression of PDK4 was significantly downregulated in HCC specimens compared with non-cancerous liver tissues (Fig. 1C). Additionally, the GEO datasets revealed that the down-regulated expression of PDK4 was observed in HCC specimens as compared with adjacent non-cancerous liver tissues (Fig. 1D). Kaplan-Meier analysis showed that HCC patients with high PDK4 expression exhibited better overall survival in the GEPIA database (Fig. 2E). Therefore, low PDK4 expression was more frequent in HCC biopsies than in their noncancerous counterparts.

Furthermore, PDK4 protein was observed in both the nucleus and cytoplasm of 7402 and 7404 cells based on an immunofluorescence assay (Fig. 1F), and PDK4 protein was also detected in the nucleus and cytoplasm of cancer cells contained in HCC clinical tissue specimens based on IHC (Fig. 1A).

### PDK4 silencing promotes the proliferation of HCC cells in vitro

Given that the data from Fig. 1 and Supplementary Table 1 demonstrated that PDK4 is significantly downregulated in HCC tissue specimens, we suspected that loss of PDK4 expression might be closely associated with HCC progression, which prompted us to perform loss-of-function experiments to further explore the effects of loss of PDK4 function on HCC cell growth by CCK-8 assay and colony formation assay. The shRNA-PDK4 specifically knocked down endogenous PDK4 mRNA (Fig. 2A) and protein (Fig. 2B) expression in both 7402 and 7404 cells. As shown in Fig. 2C, D, the results of the CCK-8 assay showed that knockdown of endogenous PDK4 by RNAi promoted cell growth in 7402 and 7404 cells. As demonstrated in the colony formation assay, shPDK4-expressing 7402 and 7404 cells formed notably more and larger colonies compared with shSCR-expressing cells (Fig. 2E, F). In summary, these findings illustrate that the loss of PDK4 expression enhances the proliferation of HCC cells in vitro.

### PDK4 knockdown enhances the motility and invasion of HCC cells

**Fig 3:**
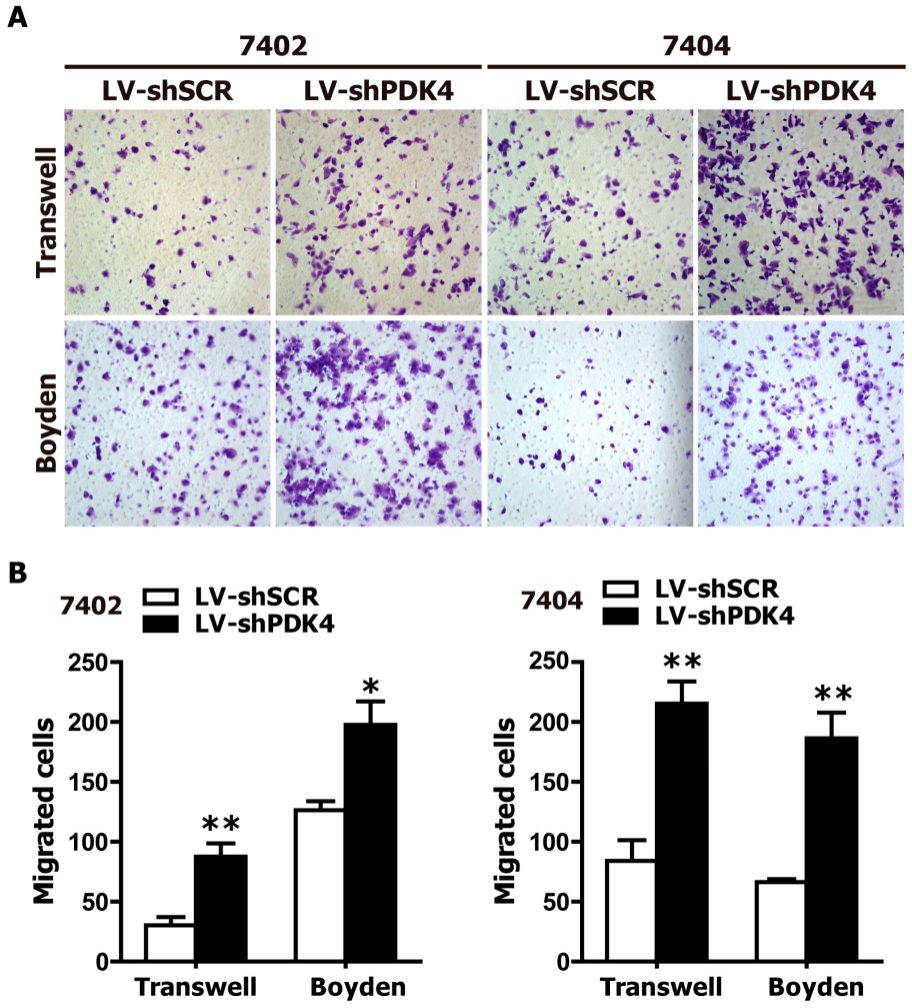
** RNAi-mediated silencing of endogenous PDK4 enhanced cell motility and invasion of HCC cells *in vitro*.** The motility and invasion activities of shSCR- and shPDK4-expressing 7402 and 7404 cells were analyzed using transwell migration and boyden invasion assays, respectively. Representative images (**A**) were presented, and the average number of migrated cells was plotted as per field of view from 3 different experiments (**B**). Statistical significance was assessed by Student's t-test (*P< 0.05 and **P< 0.01).

As PDK4 downregulation was found in the HCC tissue specimens, we suspected that PDK4 might be closely associated with the motility and invasion of HCC cells. Therefore, we also examined the effects of PDK4 silencing by RNAi on the motility and invasion abilities of HCC cells based on transwell migration and boyden invasion assays. As shown in Fig. 3, shPDK4-expressing 7402 and 7404 cells displayed significantly enhanced mobility and invasion abilities compared to those of shSCR-expressing cells. Taken together, the suppression of endogenous PDK4 expression in HCC cells promotes the migration and invasion of HCC cells.

### Silencing of endogenous PDK4 promotes the tumorigenicity of HCC cells in nude mice

**Fig 4:**
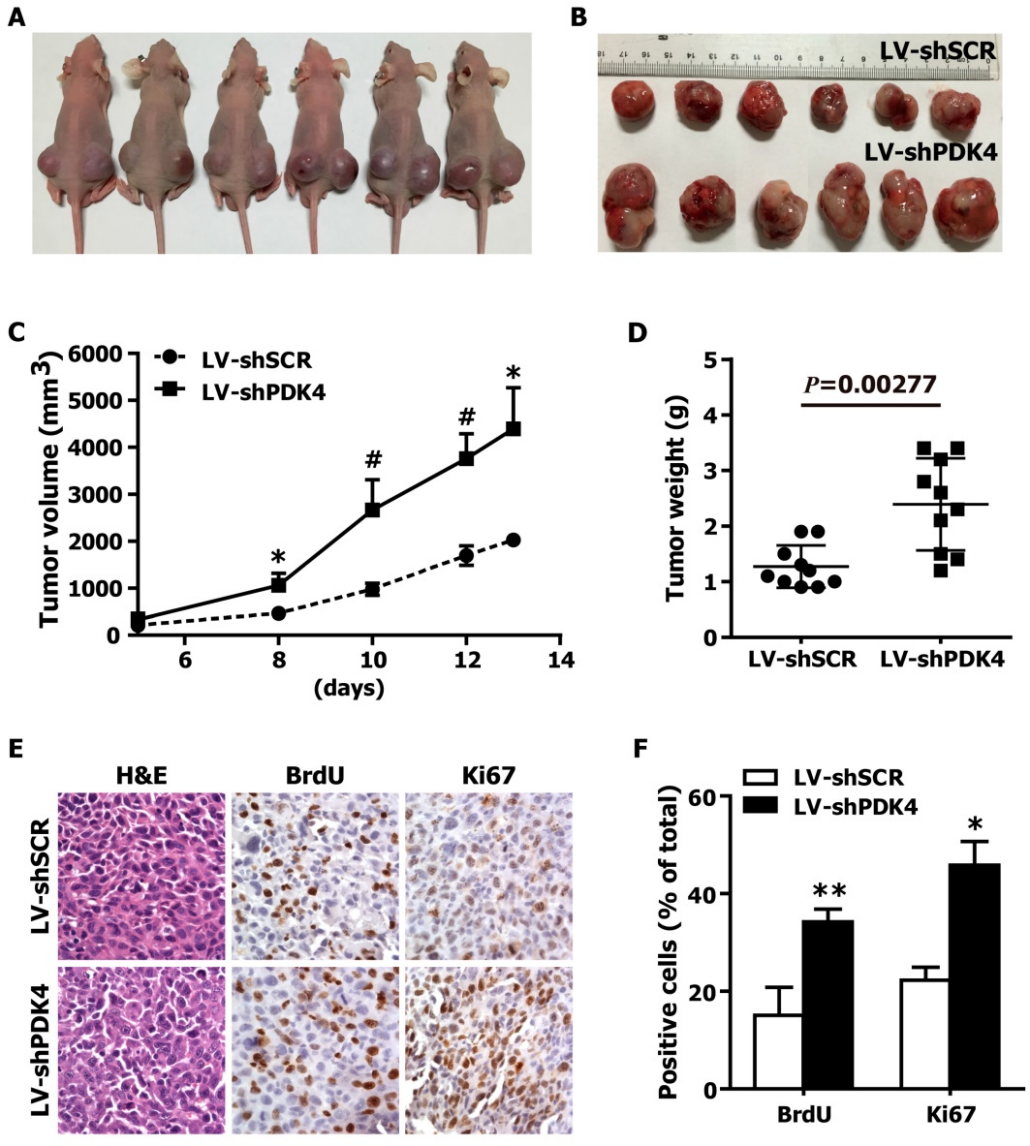
** RNAi-mediated silencing of PDK4 expression promoted tumorigenesis of HCC cells in nude mice. (A)** Representative image of 6 nude mice, which were injected with shSCR and shPDK4-expressed 7404 cells (left: LV-shSCR, right: LV-shPDK4). **(B)** Representative picture of tumors formed. **(C)** The growth curve of tumor volumes. **(D)** Tumor weight. **(E)** H&E-, BrdU- and Ki67-stained sections of transplanted tumors formed by 7404 cells. **(F)** The percentages of BrdU and Ki67 positive cancer cells were calculated by the total number of BrdU-, Ki67-positive cells over total number of cancer cells. Statistical significance was assessed by Student's t-test (*P< 0.05, **P< 0.01 and ^#^*P*< 0.001).

To further confirm the growth-promoting effects of PDK4 knockdown on HCC cells in vivo, we generated xenograft models in nude mice. shSCR- and shPDK4-expressing 7404 cells were injected subcutaneously into the dorsal flank of nude mice. The tumors became palpable 8 days after inoculation. The tumor size (Fig. 4A,B), tumor volume (Fig. 4C) and tumor weight (Fig. 4D) were significantly larger in tumors induced by shPDK4-expressing cells compared with tumors induced by shSCR-expressing cells. Additionally, the results of the immunohistochemical analysis revealed that the numbers of hyperproliferative BrdU- and Ki67-positive tumor cells from shPDK4-expressing cells were significantly greater than those of the control (Fig. 4E, F). Collectively, these findings demonstrate that depletion of endogenous PDK4 markedly accelerated tumor growth in vivo.

## Discussion

Although microarray data 17, 19 revealed that PDK4 mRNA expression is dramatically reduced in HCC biopsies, the present study was the first to demonstrate that the expression of PDK4 was significantly decreased in human HCC specimens compared to corresponding controls based on IHC and qRT-PCR. Moreover, our results from both immunofluorescence and IHC assay demonstrated that the localization of PDK4 protein was observed in both the nucleus and cytoplasm of HCC cells. More importantly, we found that PDK4 knockdown by RNAi notably promoted the proliferation, tumorigenicity, motility and invasion of HCC cells; however, the underlying mechanisms are not well elucidated. Furthermore, the previous study revealed that Erk regulation of pyruvate dehydrogenase flux through PDK4 modulated cancer cells (i.e., MCF-10A cells) proliferation 17, 19. Furthermore, PDK4 overexpression in ECM-detached cells suppressed the ErbB2-mediated rescue of ATP levels, and in attached cells, PDK4 overexpression decreased PDH flux, de novo lipogenesis and cell proliferation, suggesting a novel mechanism by which ECM attachment, growth factors, and oncogenes modulate the metabolic fate of glucose by controlling PDK4 expression and PDH flux to influence proliferation 17, 19.

Unlike the oncogenic roles of PDK1-3 in multiple human cancers, the findings from this study and those from other labs have illustrated that PDK4 plays a divergent role in various cancers, depending on different cell contexts. Evidence from studies of HCC 23, lung cancer 16, 19, ovarian cancer 20 and endometrial cancer 18 has indicated that PDK4 functions as a tumor suppressor, whereas PDK4 exerts pro-tumorigenic effects in colorectal cancer 15, prostate cancer^24^ and bladder cancer^25^. Therefore, large-scale studies are required to fully dissect the functions of PDK4 in tumor progression and the underlying mechanisms in a variety of human tumor types, which will explain why PDK4 acts as an oncogene or a tumor suppressor gene in various cancers.

Tumor suppressors are often downregulated in cancers due to their promoter DNA hypermethylation 26, 27. PDK4 was identified as a tumor suppressor because its expression was markedly diminished in human HCC clinical specimens 17, 19, and the present study showed that PDK4 silencing by RNAi resulted in significantly enhanced proliferation, tumorigenicity, motility and invasion of HCC cells. Additionally, PDK4^-/-^ livers showed increased hepatocyte proliferation, which was diminished by arsenic treatment 23. Treatment of multiple HCC cell lines with the demethylating agent 5'-aza-2'-deoxycytidine (Aza) or histone deacetylase inhibitor trichostatin-A (TSA) showed an induction of PDK4 mRNA expression in Hep3B and HepG2 cells by Aza and in Hep3B, MH97H and MH97L cells by TSA 23. Moreover, Aza decreased the methylation of PDK4 gene promoter in HepG2 cells, which was in agreement with the elevated PDK4 mRNA levels 23. Overall, the aforementioned information and data suggest that PDK4 downregulation in a cohort of HCC clinical specimens is closely related to epigenetic silencing, but this concept is not yet fully understood.

It is well known that PDK4 protein is mainly localized in the mitochondrion and peroxisome. Confocal imagining of the subcellular localization of PDK4 protein in 7404 cells (this study), huh7 cells^28^ and hepG2 cells (http://www. ptglab.com/Products/PDK4-Antibody-12949-1-AP.htm) revealed that PDK4 protein is mainly localized in the cytoplasm and is also present in the nucleus at a small level, whereas PDK4 protein is localized in the cytoplasm and nucleus of 7402 cells (this study), HeLa cells (https://www.novusbio.com/products/pdk4-antibody_nbp1-07049) and human placental trophoblasts^29^, suggesting the differently subcellular localization of PDK4 protein in various cells. These findings mean that the differently subcellular localization of PDK4 protein might play different functions in various cells.

In conclusion, we demonstrated for the first time that PDK4 plays a tumor-suppressing role in the pathogenesis of HCC. Therefore, PDK4 may be a promising therapeutic target for the treatment of advanced HCC.

## Supplementary Material

Supplementary figures and tables.

## References

[1] Ban D, Ogura T, Akahoshi K, Tanabe M (2018). Current topics in the surgical treatments for hepatocellular carcinoma. Annals of gastroenterological surgery.

[2] Brown ZJ, Heinrich B, Greten TF (2018). Mouse models of hepatocellular carcinoma: an overview and highlights for immunotherapy research. Nature Reviews Gastroenterology & Hepatology.

[3] da Motta Girardi D, Correa TS, Crosara Teixeira M, Dos Santos Fernandes G (2018). Hepatocellular Carcinoma: Review of Targeted and Immune Therapies. Journal of gastrointestinal cancer.

[4] Inarrairaegui M, Melero I, Sangro B (2018). Immunotherapy of Hepatocellular Carcinoma: Facts and Hopes. Clinical cancer research: an official journal of the American Association for Cancer Research.

[5] Nault JC, Sutter O, Nahon P, Ganne-Carrie N, Seror O (2017). Percutaneous treatment of hepatocellular carcinoma: State of the art and innovations. Journal of hepatology.

[6] Nault JC, Galle PR, Marquardt JU (2018). The role of molecular enrichment on future therapies in hepatocellular carcinoma. Journal of hepatology.

[7] Raoul JL, Kudo M, Finn RS, Edeline J, Reig M, Galle PR (2018). Systemic therapy for intermediate and advanced hepatocellular carcinoma: Sorafenib and beyond. Cancer treatment reviews.

[8] Jeoung NH (2015). Pyruvate Dehydrogenase Kinases: Therapeutic Targets for Diabetes and Cancers. Diabetes & metabolism journal.

[9] Saunier E, Benelli C, Bortoli S (2016). The pyruvate dehydrogenase complex in cancer: An old metabolic gatekeeper regulated by new pathways and pharmacological agents. International journal of cancer.

[10] Stacpoole PW (2017). Therapeutic Targeting of the Pyruvate Dehydrogenase Complex/Pyruvate Dehydrogenase Kinase (PDC/PDK) Axis in Cancer. Journal of the National Cancer Institute.

[11] Sugden MC, Holness MJ (2003). Recent advances in mechanisms regulating glucose oxidation at the level of the pyruvate dehydrogenase complex by PDKs. American journal of physiology Endocrinology and metabolism.

[12] Zhang W, Zhang SL, Hu X, Tam KY (2015). Targeting Tumor Metabolism for Cancer Treatment: Is Pyruvate Dehydrogenase Kinases (PDKs) a Viable Anticancer Target?. International journal of biological sciences.

[13] Zhang SL, Hu X, Zhang W, Yao H, Tam KY (2015). Development of pyruvate dehydrogenase kinase inhibitors in medicinal chemistry with particular emphasis as anticancer agents. Drug discovery today.

[14] Blouin JM, Penot G, Collinet M, Nacfer M, Forest C, Laurent-Puig P (2011). Butyrate elicits a metabolic switch in human colon cancer cells by targeting the pyruvate dehydrogenase complex. International journal of cancer.

[15] Leclerc D, Pham DN, Levesque N, Truongcao M, Foulkes WD, Sapienza C (2017). Oncogenic role of PDK4 in human colon cancer cells. British journal of cancer.

[16] Li G, Li M, Hu J, Lei R, Xiong H, Ji H The microRNA-182-PDK4 axis regulates lung tumorigenesis by modulating pyruvate dehydrogenase and lipogenesis. 2017; 36: 989-98.

[17] Grassian AR, Metallo CM, Coloff JL, Stephanopoulos G, Brugge JS (2011). Erk regulation of pyruvate dehydrogenase flux through PDK4 modulates cell proliferation. Genes & development.

[18] Pant A, Lee, II, Lu Z, Rueda BR, Schink J, Kim JJ (2012). Inhibition of AKT with the orally active allosteric AKT inhibitor, MK-2206, sensitizes endometrial cancer cells to progestin. PloS one.

[19] Sun Y, Daemen A, Hatzivassiliou G, Arnott D, Wilson C, Zhuang G (2014). Metabolic and transcriptional profiling reveals pyruvate dehydrogenase kinase 4 as a mediator of epithelial-mesenchymal transition and drug resistance in tumor cells. Cancer & metabolism.

[20] Sun S, Liu J, Zhao M, Han Y, Chen P, Mo Q (2017). Loss of the novel mitochondrial protein FAM210B promotes metastasis via PDK4-dependent metabolic reprogramming. Cell death & disease.

[21] Cheng X, Zhang X, Su J, Zhang Y, Zhou W, Zhou J (2015). miR-19b downregulates intestinal SOCS3 to reduce intestinal inflammation in Crohn's disease. Scientific reports.

[22] Gao F, Zhao ZL, Zhao WT, Fan QR, Wang SC, Li J (2013). miR-9 modulates the expression of interferon-regulated genes and MHC class I molecules in human nasopharyngeal carcinoma cells. Biochemical and biophysical research communications.

[23] Choiniere J, Wu J, Wang L (2017). Pyruvate Dehydrogenase Kinase 4 Deficiency Results in Expedited Cellular Proliferation through E2F1-Mediated Increase of Cyclins. Molecular pharmacology.

[24] Liu Z, Chen X, Wang Y, Peng H, Wang Y, Jing Y (2014). PDK4 protein promotes tumorigenesis through activation of cAMP-response element-binding protein (CREB)-Ras homolog enriched in brain (RHEB)-mTORC1 signaling cascade. The Journal of biological chemistry.

[25] Woolbright BL, Choudhary D, Mikhalyuk A, Trammel C, Shanmugam S, Abbott E (2018). The Role of Pyruvate Dehydrogenase Kinase-4 (PDK4) in Bladder Cancer and Chemoresistance. Molecular Cancer Therapeutics.

[26] Morgan AE, Davies TJ, Mc Auley MT (2018). The role of DNA methylation in ageing and cancer. The Proceedings of the Nutrition Society.

[27] Pfeifer GP (2018). Defining Driver DNA Methylation Changes in Human Cancer. International journal of molecular sciences.

[28] Wu J, Zhao Y, Park YK, Lee JY, Gao L, Zhao J (2018). Loss of PDK4 switches the hepatic NF-kappaB/TNF pathway from pro-survival to pro-apoptosis. Hepatology (Baltimore, Md).

[29] Liu X, Zuo R, Bao Y, Qu X, Sun K, Ying H (2017). Down-regulation of PDK4 is Critical for the Switch of Carbohydrate Catabolism during Syncytialization of Human Placental Trophoblasts. Scientific reports.

