# Supplementary material for: Loss of PDK4 expression promotes proliferation, tumorigenicity, motility and invasion of hepatocellular carcinoma cells
Source: J Cancer. 2020 May 18;11(15):4397–405. doi: 10.7150/jca.43459 (PMC7255379; doi:10.7150/jca.43459)
Supplement: Supplementary file 1 — Supplementary figures and tables. [file jcav11p4397s1.pdf]

Supplementary Figures

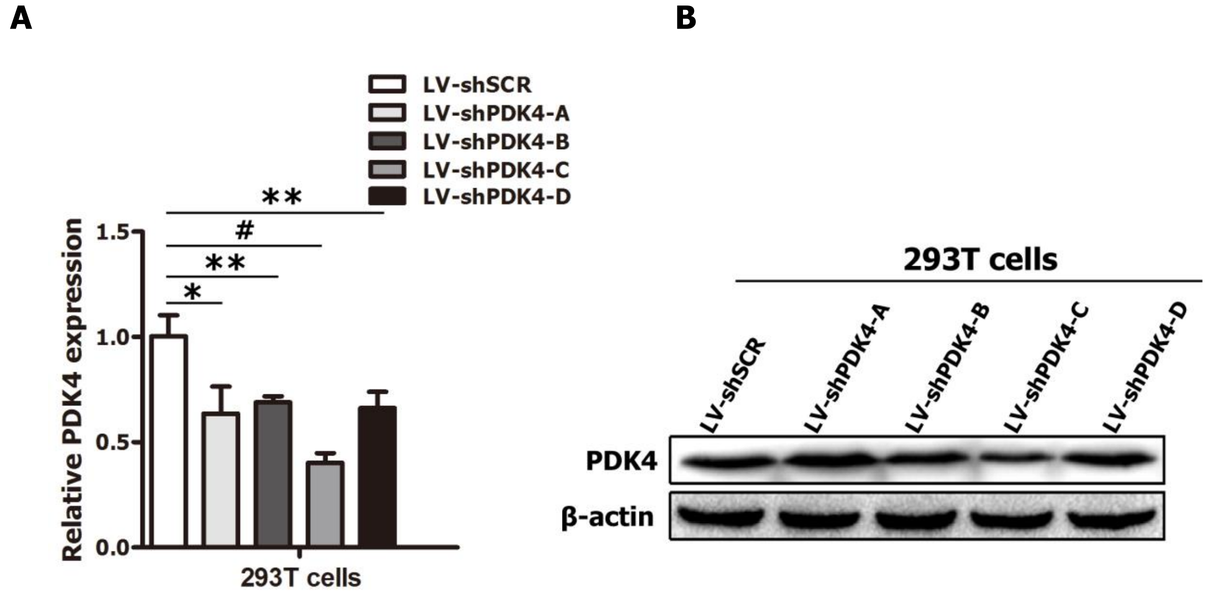

Supplementary Fig. 1. qRT-PCR analysis of PDK4 expression in 293T cells transfected with the indicated siRNA-PDK4 oligonucleotides.

In order to determine the most effective interference sequence, the vector of pLV-shPDK4-A, -B, -C or -D were transiently transfected into HEK293T cells, followed by using qRT-PCR (A) and Western blot (B) to detect the interference effects and filter out the most effective interference sequence. Our results showed that shPDK4-C is the most effective interference sequence (Supplementary Fig. 1). shPDK4-C was chosen to be used for all studies.

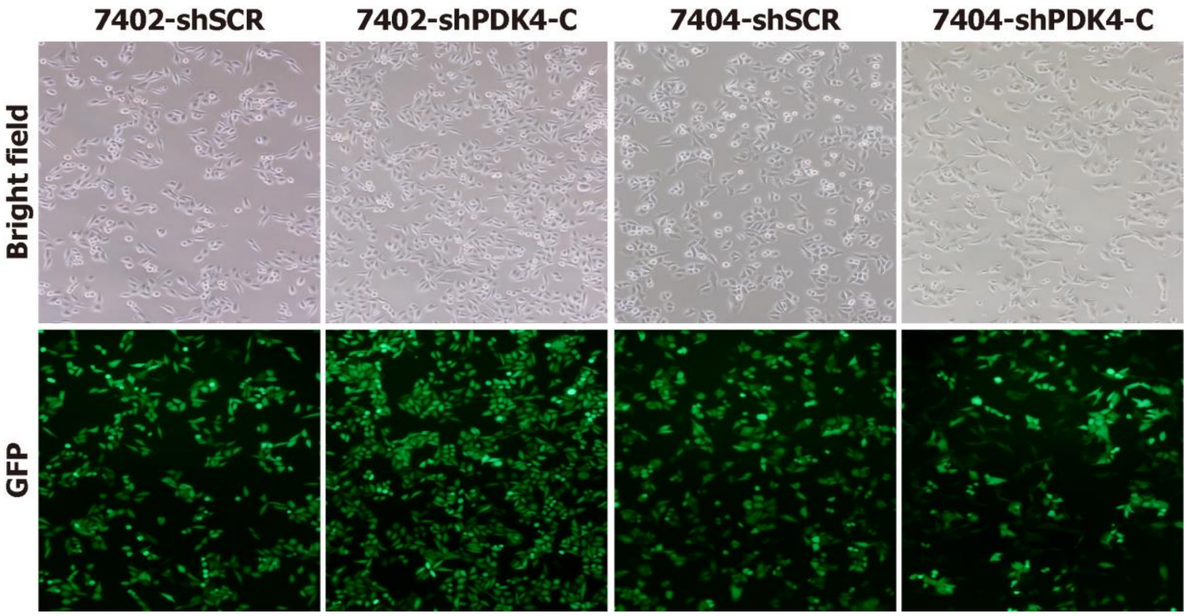

Supplementary Fig. 2. 7402 cells and 7404 cells were successfully infected by lentivirus carrying PDK4 and GFP.

Supplementary Tables

Supplementary Table 1. Expression of PDK4 in 39 adjacent non-HCC tissues and 39 HCC tissues

Variables	*n*	PDK4 expression		$\chi 2$	*p*
		Low (*n*, %)	High (*n*, %)		
Adjacent non-HCC	39	8 (20.5)	31 (79.5)	29.558	0.000
HCC	39	32 (82.1)	7 (17.9)		

Supplementary Table 2. List of antibodies and suppliers used for Western blot and immunohistochemistry

Antibody	Isotype	Suppliers
Ki67	Rabbit	Abcam
BrdU	Mouse	GE Healthcare
PDK4	Rabbit	Proteintech
β-actin	Rabbit	Cell Signaling
